# Contemporary Management Strategies of Baffle Leaks in Adults with a Failing Systemic Right Ventricle Late after Atrial Switch: A Case Series and Literature Overview

**DOI:** 10.3390/jcdd10030129

**Published:** 2023-03-17

**Authors:** Ralph M. L. Neijenhuis, Madelien V. Regeer, Frank van der Kley, Hubert W. Vliegen, Monique R. M. Jongbloed, Philippine Kiès, Martin J. Schalij, J. Wouter Jukema, Anastasia D. Egorova

**Affiliations:** 1 CAHAL, Center for Congenital Heart Disease Amsterdam Leiden, Leiden University Medical Center, 2333 ZA Leiden, The Netherlands; r.m.l.neijenhuis@lumc.nl (R.M.L.N.);; 2 Department of Cardiology, Leiden University Medical Center, 2333 ZA Leiden, The Netherlands; 3 Department of Anatomy & Embryology, Leiden University Medical Center, 2333 ZA Leiden, The Netherlands; 4 Netherlands Heart Institute, 3511 EP Utrecht, The Netherlands

**Keywords:** adult congenital heart disease, transposition of the great arteries, atrial switch procedure, mustard, senning, baffle leaks, baffle complications, (percutaneous) (trans)catheter intervention

## Abstract

Baffle leaks are a frequently encountered and often overlooked complication after the atrial switch procedure for transposition of the great arteries. Baffle leaks are present in up to 50% of non-selected patients, and while they initially may not cause clear symptoms, they can complicate the hemodynamic course and influence the prognosis in this complex patient group. A shunt from the pulmonary venous atrium (PVA) to the systemic venous atrium (SVA) can lead to pulmonary overflow and subpulmonary left ventricular (LV) volume overload, while a shunt from the SVA to the PVA can result in (exercise-associated) cyanosis and paradoxical embolism. We report three cases of baffle leaks in patients with systemic right ventricular (sRV) failure late after the atrial switch procedure. Two symptomatic patients who presented with exercise-associated cyanosis due to SVA to PVA shunting over the baffle leak underwent successful percutaneous baffle leak closure with a septal occluder device. One patient with overt sRV failure and signs of subpulmonary LV volume overload due to PVA to SVA shunting was managed conservatively, as baffle leak closure was expected to lead to an increase in sRV end-diastolic pressure and aggravation of sRV dysfunction. These three cases illustrate the considerations made, challenges faced, and necessity of a patient-tailored approach when addressing baffle leaks.

## 1. Introduction

Transposition of the great arteries (TGA) is the second most common cyanotic congenital heart defect after Tetralogy of Fallot and affects 2 to 3 out of every 10,000 live births [1]. TGA is characterized by atrioventricular concordance and ventriculo-arterial discordance; the aorta is positioned above the right ventricle (RV) and the pulmonary artery above the left ventricle (LV). In the modern era, TGA is typically managed with the arterial switch procedure to correct the ventriculo-arterial discordance. However, until around the 1980s, patients with TGA underwent the atrial switch procedure according to Mustard or Senning [2,3]. This procedure involves rerouting the systemic and pulmonary venous returns via intra-atrial baffles, resulting in a systemic right ventricle (sRV) that supplies the systemic circulation and a subpulmonary LV that supplies the pulmonary circulation.

The morphological and functional characteristics of the sRV, which are not equipped to chronically sustain the pressure of the systemic circulation, lead to a range of long-term complications. Over 50% of patients with TGA who underwent the atrial switch procedure develop symptomatic heart failure by the age of 45 years, and their 40-year survival rate varies from 82 to 90% [4,5]. While heart failure and arrhythmias are well-known late sequelae after the atrial switch operation, baffle-associated complications, such as baffle leaks and stenosis, are frequently overlooked. Baffle leaks are reported in up to 50% of non-selected and asymptomatic patients late after the atrial switch procedure [6]. Although these leaks may not directly cause symptoms, they can complicate the hemodynamic course of this complex patient group. Consequently, baffle leaks are important to be aware of, in particular in the setting of reduced systolic sRV function. A left-to-right shunt (from the pulmonary venous atrium (PVA) to the systemic venous atrium (SVA)) can lead to subpulmonary (left) ventricular volume overload and pulmonary overflow, while a right-to-left shunt (from the SVA to the PVA) can result in cyanosis and paradoxical embolism. 

The diagnosis of baffle leaks can be challenging due to the low sensitivity of transthoracic echocardiography, and might necessitate contrast echocardiography, cardiac magnetic resonance imaging (MRI), computed tomography (CT), and/or exercise testing [7,8]. The 2020 adult congenital heart disease (ACHD) guidelines of the European Society of Cardiology (ESC) recommend the closure of baffle leaks in symptomatic patients and in patients with a suspicion of paradoxical emboli (class I recommendation). Closure should be considered in asymptomatic patients with substantial ventricular volume overload (class IIa recommendation) [7]. Nonetheless, the benefits of intervention remain unclear as there is a discrepancy between a high prevalence of baffle leaks and a low prevalence of associated symptoms and related clinical deterioration [9].

This case series presents three cases of adult TGA patients with reduced systolic sRV function and baffle leaks late after the atrial switch procedure, illustrating the patient-tailored approach to the management of baffle leaks in the modern era. 

## 2. Case Descriptions

### 2.1. Patient A

A 53-year-old woman born with TGA who underwent atrial correction according to Mustard at the age of 3 years was reviewed in the outpatient clinic due to sRV failure. She had a rate adaptive atrial (AAI-R) pacemaker implanted at the age of 29 due to sick sinus syndrome, which was upgraded to a primary prevention dual chamber implantable cardioverter-defibrillator (DDD-ICD) at the age of 45. She had a history of an ambulant, uncomplicated COVID-19 infection two years ago and has since been checking her peripheral oxygen saturations (SpO2) at home. The patient now reports that SpO2 drops to 87% at ambient air during daily activities. 

**Figure 1:**
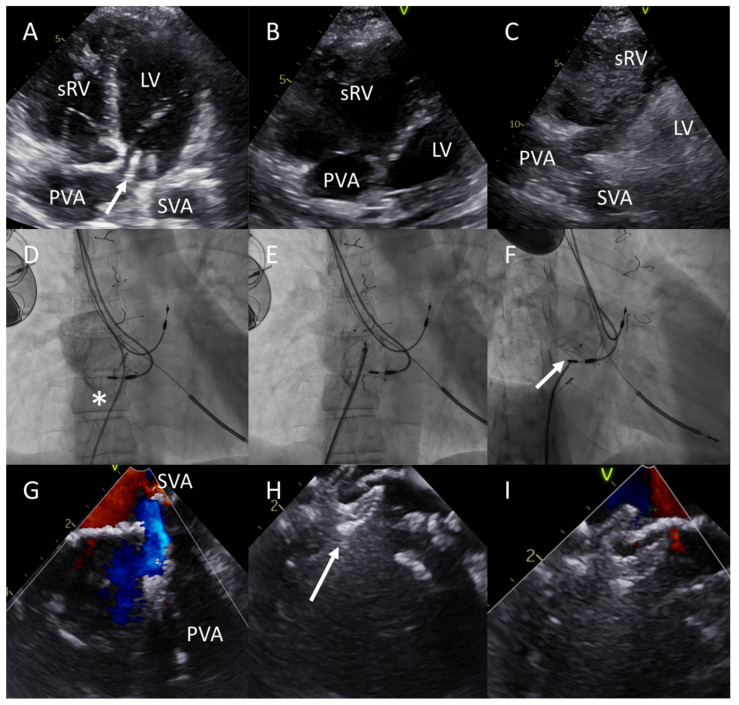
Baffle leak visualisation and closure in patient A. (**A**,**B**) Apical four chamber and subcostal views, respectively show the atrial baffle (arrow) that directs blood from the systemic venous atrium (SVA) to the subpulmonary left ventricle (LV) and from the pulmonary venous atrium (PVA) to the dilated systemic right ventricle (sRV). (**C**) Agitated saline contrast echocardiography confirms significant interatrial shunting from the SVA to the PVA during Valsalva, with bubbles filling the sRV cavity within 3 heart beats. (**D**–**F**) Anterior-posterior (**D**,**E**) and right inferior oblique (**F**) fluoroscopy projections during intracardiac echocardiography (ICE, probe indicated with an asterisk (*) in (**D**)) guided closure of the baffle leak with a 10 mm Amplatzer Septal Occluder (arrow in **F**). Note the two atrial pacing wires and an implantable cardioverter-defibrillator (ICD) lead in the LV. (**G**–**I**) ICE imaging during closure of the baffle leak. Note the shunting from the SVA to the PVA during Valsalva through the baffle defect (**G**), the discs of the Amplatzer Septal Occluder in situ (**H**, arrow) and the alleviation of the interatrial shunt (**I**).

She was in New York Heart Association (NYHA) functional class III despite pharmacological treatment with a beta-blocker, an angiotensin receptor-neprilysin inhibitor (ARNI), and a sodium-glucose cotransporter 2 inhibitor (SGLT2i). Bicycle exercise ergometry revealed an exercise capacity of 100 watts (95% of what was predicted) with a VO2 max of 19.5 mL/min/kg (66% of what was predicted) with progressive decline in SpO2 to 77% at maximal effort. Her SpO2 in ambient air was 97% at rest, and she was euvolemic. Transthoracic echocardiography (TTE) showed a poor systolic function of the dilated sRV and trivial tricuspid regurgitation. The function of the subpulmonary LV was normal (Figure 1A,B). Subsequent agitated saline contrast echocardiography showed a significant interatrial shunt from the SVA to the PVA (right-to-left) during Valsalva, and additional color Doppler examination confirmed the presence of a leak in the interatrial baffle (Figure 1C). Invasive hemodynamic evaluation showed mildly elevated pulmonary artery pressures (PAP) (26/18 mmHg, mean PAP 21 mmHg) and elevated sRV end-diastolic pressures (114/8-19 mmHg). The subpulmonary LV pressures were 40/1-9 mmHg, and no significant LV outflow tract gradient or baffle obstruction was present. A PVA to SVA shunt was present at rest (Qp:Qs 1:2.3), and an SVA to PVA shunt physiology with significant desaturation occurred during exercise as mimicked by the Valsalva maneuver in the catheterization laboratory. The patient was discussed with the heart team. Given the severity of symptomatic exercise-induced hypoxemia and the lack of PVA to SVA shunting during exercise—preventing dynamic ‘pressure relief’ for the sRV despite elevated sRV end-diastolic pressures at rest—it was decided to close the baffle leak percutaneously. 

The patient underwent a successful baffle leak closure with a 10 mm Amplatzer Septal Occluder (Abbott, Chicago, IL, USA). The procedure was performed using left femoral access under local anesthesia with fluoroscopic and intracardiac echocardiography (ICE) guidance (Figure 1D–I). No residual shunt could be detected using ICE at the end of the procedure. At 3 months follow-up, no residual shunt could be visualized on TTE, and the patient showed no desaturations below 95% at ambient air during bicycle exercise ergometry with a stable exercise capacity.

### 2.2. Patient B

A 47-year-old woman with TGA late after the atrial switch procedure according to Senning, was analyzed in the outpatient clinic due to progressive dyspnea and decreased exercise tolerance over the past year. She had a history of concomitant pulmonary valve commissurotomy, resection of an infundibular pulmonary stenosis, and ventricular septal defect (VSD) closure at the age of two years. She had two uncomplicated pregnancies at the ages of 23 and 24, respectively. A third pregnancy at the age of 26 years was terminated during the first semester due to a decline in clinical condition and persistent hypertension. In the following decades, as the sRV progressively deteriorated to a moderately reduced systolic function, she underwent a total of five atrial tachycardia ablation procedures, and a dual chamber (DDD) pacemaker was implanted due to sick sinus syndrome (with two atrial leads to enable atrial antitachycardia pacing). 

**Figure 2:**
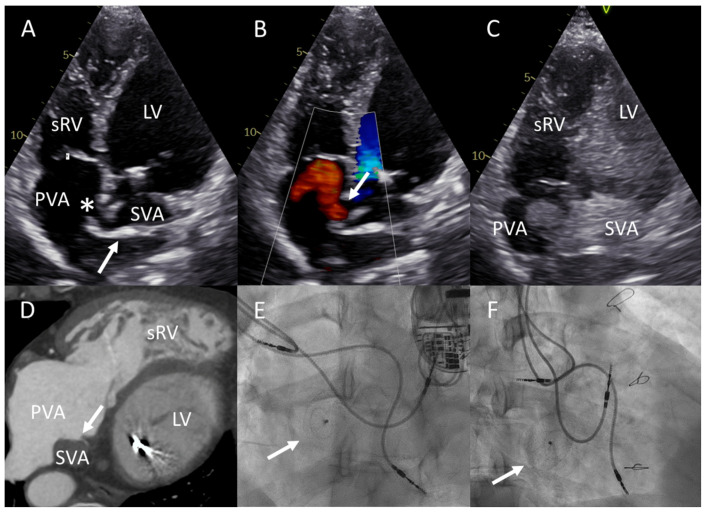
Baffle leak visualization and closure in patient B. (**A**) Apical four chamber view shows that the atrial baffle (arrow) directs systemic venous return atrium (SVA) to the subpulmonary left ventricle (LV) and the pulmonary venous return atrium (PVA) to the systemic right ventricle (sRV). The baffle leak is indicated by an asterisk (*). (**B**) Color Doppler apical four chamber view demonstrates SVA to PVA shunting through the baffle leak. (**C**) Significant interatrial shunting confirmed by agitated saline contrast echocardiography. Note the contrast opacification of the SVA and subpulmonary LV, and the jet crossing the baffle leak into the PVA. (**D**) Axial slice through a computed tomography scan at the level of the atria shows the baffle leak (arrow). (**E**,**F**) Left anterior oblique and right superior oblique fluoroscopy projections, respectively show the Occlutech Figulla Flex II Atrial Septal Defect (ASD) Occluder (arrow) projecting over the interatrial baffle. Note the presence of one ventricular (LV) and two atrial (SVA) pacemaker leads.

Upon presentation, she was in NYHA functional class III. Pharmacological treatment included a beta-blocker, an ARNI, a mineralocorticoid receptor antagonist, a loop diuretic, amiodarone, and a vitamin K antagonist. Bicycle exercise ergometry revealed a reduced exercise capacity of 80 watts (61% of what was predicted) and a VO2 max of 12.5 mL/min/kg (48% of what was predicted). SpO2 decreased from 93% at ambient air at rest to 84% at maximal effort. TTE showed a stable, moderately reduced function of the sRV, trivial tricuspid regurgitation, and preserved function of the subpulmonary LV. Suspicion of a baffle leak was raised by the apical four chamber view, which revealed systolic flow from the SVA to the PVA (right-to-left) at rest (Figure 2A,B). Significant interatrial shunting was further confirmed by agitated saline contrast echocardiography (Figure 2C). The contrast echocardiography was complicated by an air embolic transient ischemic attack, from which the patient, luckily, fully recovered. Computed tomography (CT) imaging confirmed the presence of a baffle leak of approximately 10–12 mm in diameter and showed no significant calcifications in proximity to the leak (Figure 2D). An invasive hemodynamic evaluation in euvolemia showed normal PAP (17/2 mmHg, mean PAP 7 mmHg), a wedge pressure of 4 mmHg, low sRV end-diastolic pressures (81/2–5 mmHg), and excluded any significant baffle obstruction with low gradients in the venous returns. The subpulmonary LV pressures were 30/2–4 mmHg, and no significant LV outflow tract gradient was present. The patient was discussed with the heart team; percutaneous device closure was deemed technically feasible, and recommended given the symptomatic exercise-exacerbated hypoxemia due to SVA to PVA shunting through the baffle leak.

The patient underwent a successful baffle leak closure with a 18 mm Occlutech Figulla Flex II Atrial Septal Defect (ASD) Occluder (Occlutech GmbH, Jena, Germany). The procedure was performed using left femoral access under conscious sedation and local anesthesia with fluoroscopic and ICE guidance (Figure 2E,F). No residual shunt could be detected on post-procedural TTE, and the patient reported significant functional improvement already days after the procedure. At the 3-month follow-up, her exercise capacity was stable with SpO2 ≥ 95% throughout the bicycle exercise ergometry.

### 2.3. Patient C 

**Figure 3:**
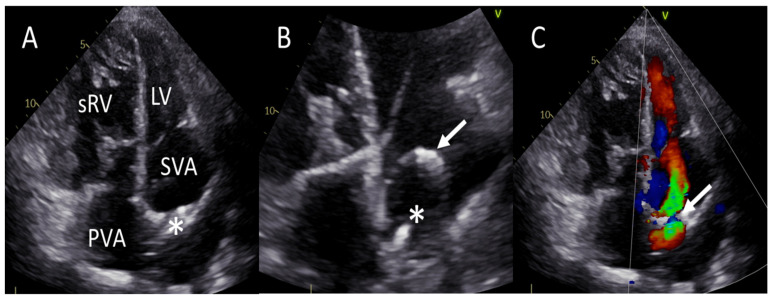
Baffle leak visualization in patient C. (**A**) Apical four chamber view shows the systemic venous return atrium (SVA), left ventricle (LV), the dilated and hypertrophied systemic right ventricle (sRV), and the pulmonary venous atrium (PVA). There is a suspicion of a baffle defect (asterisk (*) in (**A**)). (**B**) A small mobile structure suspicious of a vegetation (asterisk (*) in (**B**)) can be seen on the SVA side of the baffle defect. Note the atrial lead (arrow). (**C**) Color flow Doppler apical four chamber view demonstrates a PVA to SVA shunt over the baffle defect (arrow).

A 60-year-old woman with TGA with VSD, secundum ASD, mild infundibular pulmonary stenosis, partial anomalous pulmonary venous drainage, and a persistent left superior vena cava was evaluated in the outpatient clinic due to progressive sRV failure. She underwent the atrial switch procedure according to Mustard with VSD closure at 11 years of age, followed by surgical closure of a baffle leak at 30 years of age, and combined tricuspid valve restrictive annuloplasty with a second surgical baffle leak closure at 43 years of age. She had a history of paroxysmal supraventricular tachycardias, for which she had undergone two catheter ablation procedures. A DDD-pacemaker was implanted due to sick sinus syndrome. The system was later upgraded to a transvenous DDD-ICD due to symptomatic episodes of ventricular tachycardia, and she required chronic ventricular pacing due to symptomatic atrioventricular conduction block and bradycardia-induced (non-sustained) polymorphic ventricular tachycardia. At the age of 57 years (three years prior to the current evaluation), the patient was diagnosed with a cardiobacterium hominis endocarditis involving a small, hemodynamically not significant baffle leak and the pacemaker leads (Figure 3A,B). Her perioperative risk was deemed too high for surgical intervention, and given the good response to antibiotic therapy, she was initiated on long-term suppression therapy. 

She was now in NYHA functional class II-III despite pharmacological treatment with a beta-blocker, an ARNI, a SGLT2i, a loop diuretic, and a vitamin K antagonist. Bicycle exercise ergometry revealed a reduced exercise capacity of 50 watts (51% of what was predicted) and a VO2 max of 8.7 mL/min/kg (36% of what was predicted). SpO2 remained above 95% during exercise. TTE showed a dilated sRV with moderately to severely reduced systolic function, signs of dyssynchrony, and mild tricuspid regurgitation. The subpulmonary LV had preserved systolic function but was dilated. During diastole, there was a septal shift towards the sRV, consistent with volume overload of the subpulmonary LV. TTE also demonstrated the presence of a hemodynamically significant baffle leak with a PVA to SVA (left-to-right) shunt causing volume overload of the subpulmonary LV and contributing to the LV dilatation (Figure 3C). 

The case was discussed in the heart team. The PVA to SVA flow over the baffle leak was deemed to have an unloading effect due to the elevated sRV end-diastolic pressure. A closure of the leak was therefore expected to increase the sRV pressures and potentially aggravate systolic sRV dysfunction. Combined with the lack of cyanosis, closure of the baffle leak at this point was therefore deferred, and it was decided to first attempt to optimize the systolic sRV function. Given the pacing-induced sRV dyssynchrony observed under chronic subpulmonary LV pacing, the patient was considered to be a good candidate for epicardial sRV lead placement and cardiac resynchronization therapy. 

## 3. Discussion

In this case series, we describe the clinical considerations and contemporary management strategies for baffle leaks in adult patients with TGA late after the atrial switch procedure. The first two cases suffered from progressive sRV failure and exercise-associated cyanosis due to SVA to PVA shunting upon exertion. The baffle leaks were successfully closed through catheter intervention, eliminating the exercise-associated hypoxemia. The third patient had progressive sRV failure without significant cyanosis, yet with a PVA to SVA shunt that resulted in a volume overload of the subpulmonary LV, while “unloading” the failing sRV. The baffle leak closure was deferred, and the initial management strategy focused on optimizing the sRV function. These three cases illustrate the considerations made and challenges faced when choosing the optimal management strategy for an individual patient with a baffle leak. 

### 3.1. Prevalence and Diagnosis of Baffle Leaks 

Baffle leaks are a common complication late after the atrial switch procedure, and they are present in up to 50% of non-selected and asymptomatic patients [6]. As a significant proportion of TGA patients suffer from heart failure late after the atrial switch procedure [4], it can be difficult to suspect a baffle leak based on symptoms alone. 

The ESC and American Heart Association (AHA) ACHD guidelines state that baffle leaks can be best diagnosed routinely with (contrast) echocardiography. Transesophageal echocardiography, cardiopulmonary exercise test, and/or cardiac MRI have an additive role in the diagnosis and evaluation of baffle leaks [7,8]. In a large study investigating the prevalence of baffle leaks, only 8 out of 65 patients had a clinical suspicion prior to diagnosis. Moreover, color Doppler TTE could only accurately identify the baffle leak in 2 out of 65 patients [6], illustrating the challenges of shunt identification on routine TTE. 

In the first two cases, a clinical suspicion of a baffle leak was present prior to diagnosis. Self-registered desaturation, objectified using an in-hospital bicycle exercise ergometry (exercise-associated cyanosis), played an important role in identifying the hemodynamic consequences of a baffle defect in a group of patients with a high a priori risk. Agitated saline contrast echocardiography and/or CT were used to confirm the diagnosis in our patients. While generally considered a safe procedure, patient B suffered from a transient ischemic attack directly after contrast echocardiography due to cerebral embolization of air bubble contrast. Transient visual disturbances have previously been described after agitated saline contrast echocardiography for baffle leak diagnosis [6]. 

Cardiac MRI can provide more reliable and robust quantification of the interatrial shunt magnitude than echocardiography, although small shunts can easily be missed [7,9]. Nevertheless, it is often not feasible in the atrial switch TGA population due to a high prevalence of non-compatible cardiac implantable electronic devices and epicardial and/or abandoned leads [10]. For these reasons, a cardiac MRI could not be performed in any of the three cases described. 

Although, to the best of our knowledge, no data has been published on exercise catheterization of TGA patients with baffle defects, exercise hemodynamics have been described as being of incremental value in understanding functional limitations in patients with a Fontan circulation [11]. Right heart catheterization during exercise may therefore better reflect on the exercise-associated complaints of the TGA patients with a failing sRV and a baffle leak and provide insight into the dynamic pathophysiological changes that take place at exertion. Although at present not standard practice in the majority of centers, performing a right heart catheterization during exercise is likely to facilitate our understanding of shunting in the context of a baffle leak and heart failure, possibly unmasking a leak at an earlier stage and guiding patient-tailored treatment. 

Patients A and C underwent the atrial switch procedure according to Mustard, and patient B according to Senning. A trend towards fewer baffle leaks after the Mustard compared with the Senning procedure has previously been described [12]. As baffle leaks generally occur along the extensive suture lines surrounding the atrial baffle, one can hypothesize that this difference might be attributed to less tension on the suture lines after the Mustard technique, as a larger amount of synthetic or pericardial material is used to create the baffle. 

### 3.2. Management of Baffle Leaks 

The current AHA ACHD guidelines are less explicit in giving direct recommendations for the management of baffle leaks than the ESC guidelines [7,8]. Judging the management of the three cases in light of the ESC guidelines, cases A and B were symptomatic patients with cyanosis during exercise and therefore had a class I indication for percutaneous baffle leak closure (level of evidence: C [7]). Case C presented a challenging dilemma of closure versus conservative management. This patient presented with progressive sRV dysfunction but without specific symptoms related to her long-existing baffle leak. Eventually, dilatation of the subpulmonary LV developed due to PVA to SVA shunting, and thus percutaneous closure should be considered if feasible according to the ESC guidelines (class IIa indication, level of evidence: C [7]). 

The decision was made to manage the baffle leak conservatively in this patient, as we expected closure to lead to further elevation of sRV end-diastolic pressures and aggravation of sRV dysfunction. A potentially positive effect of a baffle leak with “ASD-like physiology” has been described in the literature. By placing a volume load on the subpulmonary LV through the PVA to SVA shunt, the interventricular septum is splinted and might support the sRV function in a manner similar to retraining the LV with a pulmonary artery band or in cases with left ventricular outflow tract obstruction/pulmonary stenosis [13]. Nevertheless, this does not seem to apply to our patient C, as there we observed a diastolic septum shift towards the sRV and a systolic shift towards the subpulmonary LV. This could actually negatively influence the sRV instead of supporting it and seems to be caused by volume-overload with a lack of pressure-overload in the subpulmonary LV.

The ESC guidelines also suggest covered stents as an option for percutaneous baffle leak closure [7]. The use of covered stents has predominantly been described for the treatment of concomitant baffle leaks and stenosis [6,10,14]. In a previous study, isolated baffle leaks were present in the minority of patients with baffle complications (17%), and a combination of occluders and/or covered stents was used to close the leaks without any major adverse events [15]. There are to date no publications comparing the two techniques, and a patient-specific, anatomy-tailored approach is essential. In our cases, no covered stents were used to avoid “jailing” of the pacing leads.

While historically baffle complications were managed surgically, perioperative mortality for surgical baffle revision after the atrial switch procedure has been reported in up to 36% of cases [16]. Percutaneous interventions are the preferred option in these adult patients, given the usually high perioperative risk due to previous thoracotomies, extensive adhesions, and concomitant heart failure.

## 4. Conclusions

Baffle leaks are present in up to 50% of non-selected patients late after the atrial switch procedure, and while they initially may not cause clear symptoms, their existence can complicate the hemodynamic course and influence the prognosis in this complex patient group. Inherent to the nature of this case series, no conclusions can be drawn to advise specific treatment strategies for patients with a baffle leak. Nonetheless, this case series demonstrates the clinical considerations in an individual, patient-tailored approach when assessing baffle leak hemodynamics in the context of sRV failure. It illustrates the feasibility of a catheter interventional management strategy using septal occluder devices for successfully alleviating exercise-associated cyanosis. Moreover, it highlights the complexity of choosing a treatment strategy in “relatively” asymptomatic patients with signs of ventricular volume overload due to PVA to SVA shunting. Although intervention should be considered in these patients, this may not always be the best option. A clear evidence-based approach is currently missing to make these challenging treatment decisions. Future research should focus on defining more robust criteria for baffle leak closure and the optimal timing window, especially for seemingly asymptomatic patients. 

## Data Availability

Not applicable.
